# Moringa Isothiocyanate-rich Seed Extract Delays the Onset of Diabetes in UC Davis Type-2 Diabetes Mellitus Rats

**DOI:** 10.1038/s41598-020-65722-6

**Published:** 2020-06-01

**Authors:** Carrie Waterman, James L. Graham, Charles D. Arnold, Kimber L. Stanhope, Jason H. Tong, Asha Jaja-Chimedza, Peter J. Havel

**Affiliations:** 1 Department of Nutrition, UC Davis, One Shields Ave, Davis, CA 95616 USA; 2 Department of Molecular Biosciences, School of Veterinary Medicine, UC Davis, USA; 3 Department of Plant Biology, Rutgers University, 59 Dudley Rd, New Brunswick, NJ 08901 USA

**Keywords:** Drug discovery, Metabolic disorders

## Abstract

Moringa seeds have been used traditionally in the management of type 2 diabetes mellitus (T2DM) and contain potent bioactive isothiocyanates. This study evaluated the efficacy of an isothiocyanate-rich moringa seed extract in delaying the onset of T2DM in UC Davis T2DM rats, a well validated model which closely mimics T2DM in humans. Rats were separated into three groups; control, moringa seed extract at 0.4%, and a weight matched group. Rats were fed respective diets for 8 months, during which energy intake, body weight, the onset of diabetes circulating hormones, metabolites and markers of inflammation and liver function, and were monitored. The MS group had a significantly slower rate of diabetes onset p = 0.027), lower plasma glucose (p = 0.043), and lower HbA1c (p = 0.008) compared with CON animals. There were no significant differences in food intake and body weight between all groups. This study demonstrated MS can delay the onset of diabetes in the UC Davis T2DM rat model to a greater extent than moderate caloric restriction (by comparison to the WM group). The results support its documented traditional uses and a bioactive role of moringa isothiocyanates and suggest the potential efficacy for moringa supplementation for diabetes management in populations at risk for T2DM.

## Introduction

Moringa (*Moringa oleifera*, Lam.) is a plant that has been used for centuries in South East Asia as both a food and as medicine. Its therapeutic applications include managing blood sugar levels and other chronic diseases related to inflammation and manifestations of metabolic syndrome^1–3^. This study aimed to provide further scientific support for the use of moringa, and it’s suggested bioactive isothiocyanate, in the prevention and treatment of diabetes; both validating traditional uses and paving the way for future clinical studies.

Moringa seeds are used for production of oil, water purification, and traditionally chewed for the management of diabetes and other ailments^2^. The seeds produce bioactive phytochemicals including 4 (α-L-rhamnosyloxy)-benzyl isothiocyanate (MIC-1) which has been shown to have potent anti-inflammatory activity; specifically attenuating expression of inflammatory cytokines: inducible nitric oxide synthase (iNOS) and interleukin-1 beta (IL-1β); and reduced production of nitric oxide (NO) and tumor necrosis factor alpha (TNF-α) in cultured macrophages at low micromolar concentrations^4^. MIC-1 was also shown to significantly reduce gluconeogenesis and glucose-6-phosphatase (G6P) expression in liver cells at similar concentrations^5^.

Studies in human subjects investigating the effects of moringa in patients with diabetes have been limited to testing only leaf extracts and demonstrated decreased fasting and post-meal blood glucose levels by 28% and 26%, respecitvely^6^. However bioactive constituents of the leaf were not determined.

While no clinical trials have been conducted with moringa seeds or seed extracts, in animal studies, in an alloxan-induced type-1 diabetes rat model moringa seed extract was shown to lower blood glucose levels^7^. Supplementation of streptozotocin-induced diabetic rats with moringa seed extract at 50 and 100 mg/day also lowered fasting blood glucose, lipid peroxide, glycated hemoglobin (HbA1c), plasma immunoglobulin (IgA and IgG), and the inflammatory cytokine interleukin-6 (IL-6). The extracts increased antioxidant activity, measured in catalase, reduced glutathione (GSH), and superoxide dismutase (SOD)^8^. While promising, both these models of chemically induced pancreatic ß-cell destruction are primarily used to evaluate treatments for type 1 diabetes^9^. Additionally, phytochemical composition of these extracts was not determined, and therefore not possible to identify the bioactive components.

A moringa seed extract (MS) optimized to contain 35–47% of the primary bioactive component, MIC-1, was previously developed, profiled^10^, and evaluated for safety and toxicity in rats^11^. The MS was also tested for mitigation of ulcerative colitis^12^ and anti-diabetic effects^13^.

In this current study, we put MS to a more rigorous evaluation for T2DM treatment. UC Davis Type 2 diabetes mellitus (UCD T2DM) rats were developed as an improved model of T2DM which more closely mimics the pathophysiology and temporal development of the disease in humans^14,15^. Rats were developed by crossing obese, insulin resistant Sprague-Dawley rats with diabetic Zucker fatty-lean rats. The resulting polygenic animal model exhibits insulin resistance, deficits in pancreatic β-cell function and altered islet morphology, but retains normal leptin signaling^16^ (ref, Cummings *et al*., PNAS 2011), and also exhibits glycosuria, polyuria, and hyperphagia – all more similar to the symptoms of T2DM in humans than the high-fat fed mouse model and other rat models of the disease. Furthermore, UCD T2DM rats develop overt diabetes, defined by two consecutive non-fasting blood glucose measurements >250 mg/dl, at an average age of 6 months, whereas high-fat fed mouse models typically only develop insulin resistance without overt hyperglycemia/diabetes. In this study, the effects of MS supplementation were compared with a control group (CON) and weight-matched group (WM) to evaluate the role MS might have by limiting food intake in the rats.

## Results

### MS delayed the onset of T2DM

**Figure 1:**
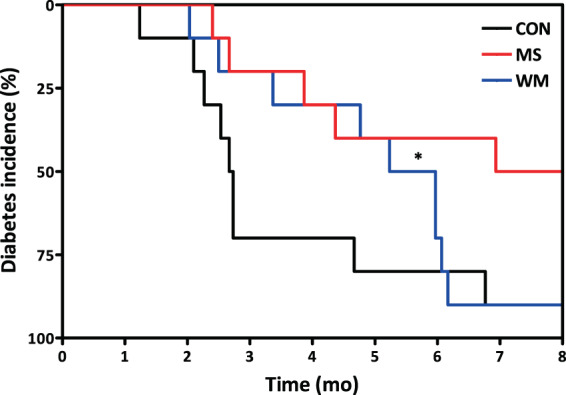
Kaplan–Meier analysis of diabetes incidence in control (CON; n = 10), MS-supplemented (MS; n = 10), and weight matched (WM; n = 10) animals.

UCD T2DM rats supplemented with MS in their diet exhibited a delay in the onset of diabetes compared with CON (p = 0.027), but not significantly different from the WM group (Fig. 1). By three months post intervention 70% of the CON rats had developed diabetes, whereas only 20% of the MS and WM had developed the disease. By seven months post intervention 90% of the CON and WM rats had developed diabetes, whereas only 40% of the MS had developed the disease.

### MS did not affect body weight or food intake

**Figure 2:**
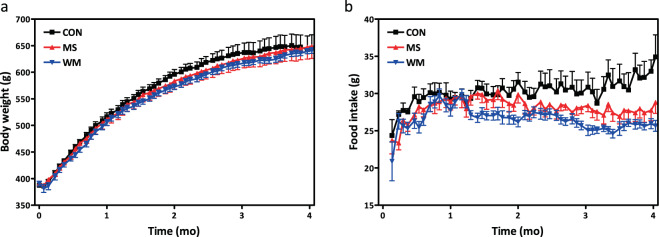
Body weight (**a**) and food intake (**b**) in control (CON; n = 10), MS-supplemented (MS; n = 10), and weight matched (WM; n = 10) animals.

MS supplementation added into the chow diet did not result in significant differences in body weight compared with the CON and WM groups (Fig. 2a). Food intake was also not significantly different between all groups (Fig. 2b), although the CON began to consume slightly more food around 10 weeks when more animals in this group had developed diabetes accompanied by diabetic hyperphagia. Overall, the WM rats consumed an average of ~6% less food/calories than the MS treated animals.

### MS lowered fasting glucose and HbA1c levels over four months. Plasma insulin concentrations differed in control vs MS

**Figure 3:**
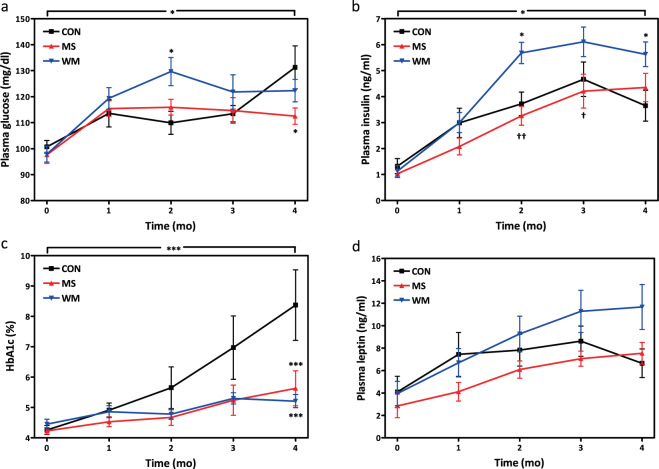
Plasma levels of fasting glucose (**a**), insulin (**b**), HbA1c (**c**), and leptin (**d**) over 4 months in control (CON; n = 10), MS-supplemented (MS; n = 10), and weight matched (WM; n = 10) animals.

Fasting glucose and HbA1c concentrations were lower in MS rats over the course of the four months compared with the control group (p = 0.043, p = 0.008, respectively) and remained similar to the WM group (Fig. 3a,c). In contrast, fasting plasma insulin concentrations in the MS group were similar to control but lower than in WM animals (Fig. 3b; p = 0.007) over the four months. Plasma leptin concentrations were not significantly different between groups (Fig. 3d).

### MS moderately improved glucose tolerance and insulin dynamics in the oral glucose tolerance test (OGTT)

**Figure 4:**
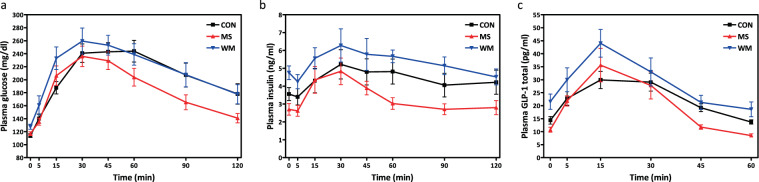
Plasma glucose (**a**), insulin (**b**), and GLP-1 (**c**) concentrations during the oral glucose tolerance tests in control (CON; n = 10), MS-supplemented (MS; n = 10), and weight matched (WM; n = 10) rats.

Although not statistically significant, mean peak levels of glucose tended to be lower and glucose appears to decrease more quickly during to 60–120-minute time points of the OGTT in the MS-treated group compared with CON and WM. (Fig. 4a). Similarly, fasting plasma insulin concentrations and insulin levels following oral glucose administration were consistently, but not significantly, lower in the MS group than CON and WM (Fig. 4b). The percent change from baseline to peak insulin for OGTT was higher in the MS group compared with the CON and WM groups, however it was not significant with a one factor ANOVA. No significant differences were observed in glucagon-like peptide-1 (GLP-1) responses to oral glucose administration between the treatment groups (Fig. 4c).

### MS did not significantly influence TC or TG. Adiponectin levels differ in control vs MS

**Figure 5:**
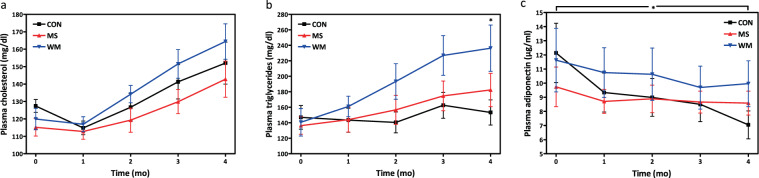
Total fasting cholesterol (**a**), triglycerides (**b**), and adiponectin (**c**) over 4 months in control (CON; n = 10), MS-supplemented (MS; n = 10), and weight matched (WM; n = 10) animals.

MS did not significantly affect plasma cholesterol or triglyceride levels (Fig. 5a,b), although a trend towards higher TG levels in the WM group was observed. A significant percent decrease from baseline (%∆ CON = −33.7 ± 8.4%) was observed for plasma adiponectin concentrations in the CON group. MS treatment appears to attenuate the decrease of circulating adiponectin observed with the progression of diabetes (Fig. 5c; %∆ MS −1.9 ± 10.7%, p = 0.011).

### MS did not increase the liver enzymes: alanine aminotransferase (ALT) and aspartate aminotransferase (AST)

**Figure 6:**
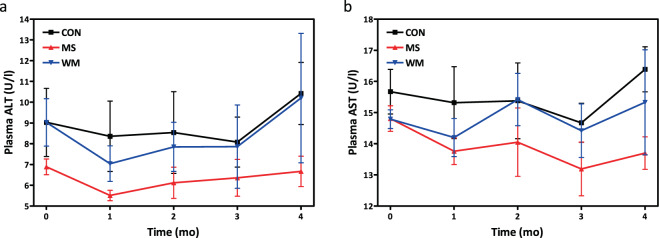
Plasma levels of fasting ALT (**a**) and AST (**b**) over 4 months in control (CON; n = 10), MS-supplemented (MS; n = 10), and weight matched (WM; n = 10) animals.

No significant differences in plasma concentrations of the liver enzymes: ALT (Fig. 6a) or AST (Fig. 6b) were observed between groups. Importantly, MS supplementation was not associated with an increase of these liver enzymes from baseline.

### MS did not significantly alter plasma levels of inflammatory cytokines KC/GRO or TNF-α

**Figure 7:**
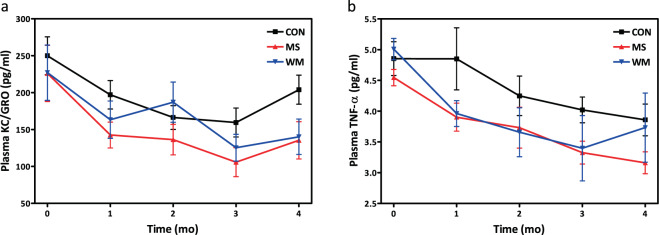
Circulating concentrations of fasting KC/GRO (**a**) and TNF-α (**b**) over 4 months in control (CON; n = 10), MS-supplemented (MS; n = 10), and weight matched (WM; n = 10) animals.

No significant differences were observed between MS and CON or MS and WM, however plasma concentrations of both cytokines tended to decrease from baseline levels over time in all three groups of animals (Fig. 7a,b). MS rats in general exhibited the lowest mean values of both cytokines throughout the study.

### Plasma corticosterone levels did not vary significantly between groups

**Figure 8:**
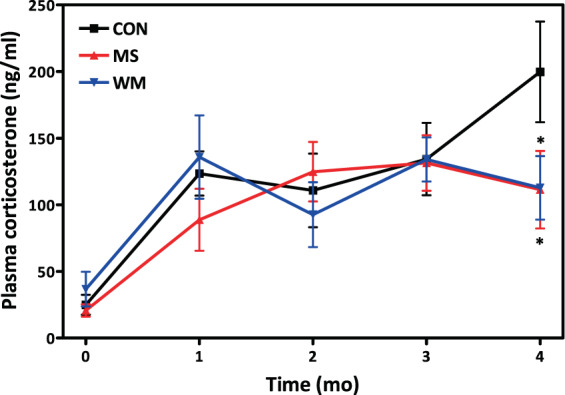
Plasma fasting corticosterone levels over 4 months in control (CON; n = 10), MS-supplemented (MS; n = 10), and weight matched (WM; n = 10) animals.

Plasma concentrations of the glucocorticoid hormone corticosterone were measured to determine whether stress might be a contributing factor to elevated levels of glucose, insulin presented in Figs. 3 and 4. The MS and WM groups had significantly lower levels of plasma corticosterone compared to CON at the 4 month timepoint, however the difference was not significant for the overall length of study (Fig. 8).

## Methods

### Animals

UCD T2DM rats were bred in the colony managed by Dr. Havel’s laboratory and housed in the Department of Nutrition animal facility at the University of California, Davis. The animals were maintained on a 14/10 h light–dark cycle. Food intake and body weight were measured three times per week. For the WM group, the animals were fed a daily ration of food in the morning and evening with each individual rat receiving an amount of food calculated to equalize the % BW gain to the mean % BW gain of the MS group. Therefore, each individual WM rat received a different amount of food, i.e. a heavier rat was provided with more food compared with a lighter rat, such that % BW gain would be the same in both groups. Non-fasting blood glucose was monitored every week with a glucose meter (One-Touch Ultra, LifeScan, Milpitas, CA, USA). Onset of diabetes was defined as a non-fasted blood glucose value above 13.9 mmol/l (250 mg/dl) for two consecutive weeks, and measurements were performed for 8 months or for up to 3 months after the onset of diabetes in the animals that developed diabetes prior to 8 months of age. The experimental protocols were approved by the University of California, Davis Institutional Animal Care and Use Committee; all experiments were performed in accordance with applicable guidelines and regulations. Starting at 2 months of age, prediabetic male animals were matched for mean body weight and divided into three groups: CON (n = 10), MS (n = 10), and WM (n = 10).

### Extract and diets

MS extract was prepared and analyzed as previously described^10^. In brief, moringa seeds were ground to a powder and incubated with water at a 1:3 ratio for 2 h at 37 °C. Ethanol was then added at 4 times the volume of water. The mixture was then filtered and dried by rotary evaporation and freeze-dried. Proximate nutritional analysis reported MS to contain 13.92% moisture, 1.76% fat, 0.6% fiber, 4.01% and 22.54% carbohydrates^10^. Phytochemical characterization was performed by mass spectrometry and determined MS to contain 46.7% MIC-1, the most dominant component^10^. Additional phytochemicals tentatively identified in MS, and their relative abundance to MIC-1 written in parenthesis (with MIC-1 being arbitrarily set at 100), included sucrose (10), malic acid (5), glucosinolates (5), catechin/epicatechin and procyanidin dimer (<0.5), niazirin (1), niazimicin (3), palmitic acid and 2-hydroxy behenic acid (2) and oleic acid (5)^10^.

MS was mixed with ground rodent chow (2018 Teklad Global, Envigo Indianapolis IN) to comprise 0.4% of the diet w/w. The CON and WM groups were fed the same ground chow that was used for the MS treatment. As food intake increased over the course of the experiment, the animal’s average consumption was 202 mg/kg/day of MS and 94 mg/kg/day of MIC-1. An initial palatability study was conducted for three weeks to determine that rats would consume the MS when mixed with the chow diet. Blood samples were collected from the tail into EDTA coated tubes after a 13 h fast monthly starting at 2 months of age and for four months after treatment began (time points: 0, 1, 2, 3 and 4 months). Tubes were centrifuged at 1500×G and 4 °C for 10 min and the plasma was frozen at −80 °C and later used for measurements of glucose, insulin, total cholesterol, triglycerides, adiponectin, leptin, AST and ALT. The OGTT was performed as previously described^14^. Rats were fasted overnight and gavaged with a 1 g/kg dose of a 50% dextrose solution. Samples were collected for the measurement of glucose insulin, and GLP-1 over 120 min.

### Assays

Plasma glucose and cholesterol were measured with enzymatic colorimetric assays (Fisher Diagnostics, Middletown VA, USA). Plasma triglycerides, ALT, and AST were measured with enzymatic colorimetric assays (Millipore Sigma, Burlington MA USA). HbA1c was measured with an enzymatic assay (Diazyme, Poway, CA USA). Plasma insulin, leptin, GLP-1, and adiponectin were measured with electrochemiluminescent immunoassays (Meso Scale Discovery, Rockville, MD USA). Corticosterone was measured with a radioimmunoassay (MP Biomedicals, Orangeburg, NY USA).

### Data analysis

Analyses focused on contrasting the MS treatment group with each of the two comparison groups, specifically MS vs CON and MS vs WM animals. Diabetes onset was analyzed by log-rank testing of Kaplan–Meier survival curves. Mixed model ANCOVA was used to account for the dependence of repeated measures on the same subject while evaluating plasma concentrations of glucose, insulin, HbA1c, leptin, total cholesterol, triglycerides, adiponectin, AST, ALT, KC/GRO, and TNF-α over time. The interaction term between time and treatment group was used to assess whether changes in concentrations differed by treatment group over time. Error bars represent 95% confidence intervals.

## Discussion

MS supplementation significantly delayed the onset of diabetes in the UCD T2DM rat model, and in a similar fashion to modest food restriction (WM group). It has been well established that a calorically restricted diet can delay the onset of diabetes and other age-related diseases^17–21^, including studies in the UCD T2DM rat model^14,22,23^. However, long-term compliance with caloric restriction can be challenging for most people to maintain^24^. Therefore, MS supplementation may mimic some of the effects of caloric restriction, without a person having to intentionally restrict their dietary intake. Furthermore, MS supplementation delayed the onset of diabetes in this rat model by at least 4–5 months which would be equivalent to a delay of diabetes onset of approximately 10–15 years in a human life-span^25^, thus significantly improving both quality of life and longevity. The results from the OGTTs in animals supplemented with moringa, (improved glucose disposal, lower fasting insulin levels, and a trend towards greater nadir to peak insulin responses to oral glucose) are compatible with a metabolic profile that would lead to a delay in the onset of diabetes.

The delay of diabetes onset is reflected by lower HbA1c levels in the MS supplemented and WM UCD-T2DM rats than in the *ad libitum* fed CON animals in which HbA1c increased progressively as more of these animals developed overt diabetes. The WM group exhibited impaired glucose tolerance, higher fasting and post-glucose insulin, and GLP-1 concentrations during the OGTT than the MS and CON groups. It is possible the modest restriction and likely the alterations in feeding patterns may have resulted in some degree of stress in the WM animals resulting in insulin resistance. This is also suggested by the higher longitudinal fasting insulin concentrations during the study in the weight-matched group (Fig. 4b). While overall plasma corticosterone concentrations (an indicator of stress) during the study were not significantly elevated in the WM group compared with the MS and CON groups (Fig. 8), increased stress cannot be ruled out as a contributing factor to these differences between the WM and MS groups. Plasma liver enzyme (ALT and AST) concentrations were measured to assess whether MS administration might induce hepatotoxicity. There were no significant differences in ALT or AST between treatment groups, although average values for the MS group tended to be the lowest, indicating MS treatment is unlikely to be hepatotoxic at the levels administered. Results for hepatic function are consistent with findings from the study with moringa seed extract in streptozotocin-induced diabetic rats which found no between group differences in ALT and AST^8^. Moringa’s effects on liver function may be worth further exploration for therapeutic effects. Importantly, fasting plasma glucose and Hb1AC concentrations in MS treated animals were significantly lower over the course of the experiment and consistent with the observed delay in the onset of diabetes in the MS group. Although previous studies in humans supplemented with moringa leaf powder have reported significant reductions of total and low-density lipoproteins (LDL) cholesterol^6,26^, MS supplementation did not significantly reduce total plasma cholesterol or triglycerides in this study. It is possible, bioactive compounds, such as polyphenols, present in the leaf powder but not in the seed extract, are involved in the lipid lowering effects of moringa. Further studies are planned to directly compare the efficacy of seed and leaf extracts and further elucidate MIC-1’s mechanisms of action in various tissues. The slight, but non-significant, reduction of inflammatory markers (KC/GRO and TNF-α) is consistent with previous evaluation of MS in the high-fat-feed mouse model^13^. However, the previous study did demonstrate anti-inflammatory activity from MS via activation of the nuclear factor erythroid 2-related factor 2 (Nrf2) pathway.

Overall, these results support traditional uses of moringa and suggest the plant may be a viable source of bioactive compounds effective in the management of T2DM. The use of moringa as an alternative or complementary medicine is particularly relevant in areas of the world lacking in affordable access to modern pharmaceutical agents. Rapid growth and cost efficiency of moringa cultivation and use in regions susceptible to climate changes and drought make it an especially interesting plant for further translational research. Results from currently available clinical trials in humans remain quite limited at this time. Data from this current study, conducted in a translationally relevant animal model of T2DM, indicate additional studies are warranted in order to better evaluate the potential of moringa and its bioactive components in the global prevention and management of chronic metabolic disease, particularly type-2 diabetes.
